# Comparative Analysis of Verapamil Pharmacokinetics: Evaluating the Impact of Simple Suspension and Crushing Administration Methods

**DOI:** 10.3390/jcm13195969

**Published:** 2024-10-08

**Authors:** Sumito Kumagai, Takehiko Sambe, Keita Shibata, Takuya Mizukami, Hokuto Morohoshi, Kakei Ryu, Taigi Yamazaki, Sachiko Takenoshita, Shunsuke Matsukawa, Saki Goibuchi, Naoki Uchida, Naomi Kurata, Noriko Hida

**Affiliations:** 1 Department of Clinical Pharmacology, Graduate School of Medicine, Showa University, Tokyo 157-8577, Japan; sumito.kuma@med.showa-u.ac.jp (S.K.); mizukamit@med.showa-u.ac.jp (T.M.); ryu-k@med.showa-u.ac.jp (K.R.); s3take@med.showa-u.ac.jp (S.T.); nuchida@med.showa-u.ac.jp (N.U.); n.hida@med.showa-u.ac.jp (N.H.); 2 Showa University Clinical Research Institute for Clinical Pharmacology and Therapeutics, Tokyo 157-8577, Japan; hokuto-n@med.showa-u.ac.jp (H.M.); t.yamazaki@cmed.showa-u.ac.jp (T.Y.); 3 Department of Pharmacology, Graduate School of Pharmacy, Showa University, Tokyo 142-8555, Japan; kshibata@pharm.showa-u.ac.jp (K.S.); s.watanabe@cmed.showa-u.ac.jp (S.G.); 4 Pharmacological Research Center, Showa University, Tokyo 157-8555, Japan; 5 Department of Hygiene, Public Health and Preventive Medicine, Graduate School of Medicine, Showa University, Tokyo 157-8555, Japan; 6 Department of Clinical Research and Development, Graduate School of Pharmacy, Showa University, Tokyo 142-8577, Japan; 7 Department of Pharmaceutics, Graduate School of Pharmacy, Showa University, Tokyo 142-8555, Japan; gp23-s020@pharm.showa-u.ac.jp; 8 Department of Social Pharmacy, Graduate School of Pharmacy, Showa University, Tokyo 142-8555, Japan; kuratan@cmed.showa-u.ac.jp; 9 Department of Clinical Nutrition and Metabolism, Graduate School of Pharmacy, Showa University, Tokyo 142-8555, Japan

**Keywords:** simple suspension method, tablet crushing, verapamil, pharmacokinetics, oral administration

## Abstract

**Background/Objective:** It is not uncommon for elderly patients to experience difficulties with feeding and swallowing. In the simple suspension method, tablets are dissolved and suspended in warm water without prior crushing or decapsulation, and then administered via a tube. Despite the prevalence of this method, the pharmacokinetics of suspended tablet dosage forms remain poorly understood. **Methods:** Verapamil was employed in dissolution tests following both the simple suspension and crushing methods. A pharmacokinetics study was conducted on healthy adult males. **Results**: The resultant dissolution profiles from the two methods exhibited notable dissimilarities. Drug release from the crushed product commenced earlier than that from the simple suspension and intact tablet. Furthermore, the area under the curve for verapamil during the initial 24 h period was 1.7 and 1.3 times greater in the crushed and simple suspension groups, respectively, than in the tablet group. **Conclusions**: The crushing and simple suspension methods are safe techniques for administering medications to patients with dysphagia, thereby preventing aspiration. Nevertheless, the processing of medications may result in an increased frequency of adverse effects. It is recommended that the processing of medicines prior to administration be avoided.

## 1. Introduction

In Japan, the birth rate is declining, and the population is rapidly aging. By 2025, the cohort of individuals born during the post-war baby boom will have reached the age of 75, marking the advent of a super-aged society [1]. To develop a medical and long-term care delivery system that can meet the rapidly increasing medical and long-term care needs of the growing elderly population, the Ministry of Health, Labour and Welfare (MHLW) has included matters related to home medical care in its medical plan since the 2013 fiscal year and promotes the differentiation and coordination of hospital bed functions. A majority of patients receiving tube feeding are those admitted to medical treatment facilities, with long-term care facilities accommodating patients with critical medical needs [2].

It is recommended that tube feeding be initiated as early as possible in older individuals when dysphagia is observed [3]. Furthermore, the necessity for tube feeding is considerable in the nutritional treatment of older patients with dysphagia. When administering medications to patients undergoing tube feeding, if liquid formulations are not an option, alternative methods must be employed, including the crushing of tablets or decapsulation of capsules. Subsequently, the powder is suspended in water prior to tube feeding [4]. The administration of food and drugs through a feeding tube is common practice in clinical settings. However, the administration of medications through a feeding tube is not always an effective or safe method and can involve any number of problems. These possible problems include interactions with other drugs, alterations of how the body absorbs the medication, decreased absorption due to interactions with nutrients, changes to how the body processes the medication, and sorbitol, which is used as a suspending agent and can cause osmotic changes. Additionally, the tube itself can become blocked [5]. The process of crushing or decapsulating intact dosage forms presents several challenges. One such challenge is the potential loss of the main drug due to adhesion to the pestle, mortar, automatic dispensing machine, or dispensing paper during the crushing and dispensing process Additionally, dispensing personnel may be exposed to the drug, which could lead to adverse effects. Furthermore, alterations to the drug’s dosage form may compromise the original design intent and alter the pharmacokinetics. Moreover, tube blockage by the injected drug represents a significant medical challenge [6].

The simple suspension method was devised to overcome these shortcomings. In this technique, tablets or capsules are disintegrated and suspended in warm water (55 °C) without crushing or decapsulation and administered via a nasogastric tube, gastrostomy, or enterostomy [6]. Although the simple suspension method has been employed in numerous medical institutions, its impact on drug pharmacokinetics has not been adequately investigated in comparison to those of tablets taken intact or after crushing. Previous studies on the simple suspension method have primarily focused on the formulation characteristics and stability of the drug, with few studies evaluating the effect of the drug administered to an actual patient or the safety of administration.

The administration of medicines via the simple suspension method facilitates the passage of most tablets and capsules through the feeding tube, thereby preventing tube clogging [7,8,9]. However, it is important to exercise caution, as the recovery rate of certain drugs, such as aspirin, after passage through a feeding tube may be reduced [10]. In general, the simple suspension method is known not to result in instability issues for most drugs and to have a high recovery rate [8,9,11]. It is possible that some drugs may undergo chemical decomposition when temporarily suspended with other drugs or solutions. Conversely, the act of crushing tablets or decapsulating capsules has the potential to alter the absorption of drugs, which could ultimately result in an overdose or a reduction in efficacy [12,13,14,15]. In particular, crushing sustained-release or enteric-coated formulations can lead to alterations in the rate of drug release, which may ultimately result in an increased risk of overdose or a reduction in efficacy [14,15]. If the crushing method is not properly executed, the efficacy of the pharmaceutical agent may be diminished, and the patient may experience adverse effects [12,13,16].

Verapamil hydrochloride (Vasolan^®^) is a pharmaceutical agent indicated for the treatment of tachyarrhythmia, angina pectoris, and myocardial infarction. It exhibits an ester structure, and its International Union of Pure and Applied Chemistry (IUPAC) nomenclature is (RS)-2-(3,4-dimethoxyphenyl)-5-[2-(3,4-dimethoxyphenyl)ethyl-methyl-amino]-2-(1-methylethyl)pentanenitrile [17]. Previously, it was prescribed as an antihypertensive for chronic use, and it is thus highly probable that it is processed in elderly care facilities, including as a simple suspension and crushed. The stability of Vasolan^®^ tablets was investigated following crushing and processing into a simple suspension; the recovery rate of the main drug in the crushing method was found to be significantly lower than that in the simple suspension method [8].

In general, ester bonds are susceptible to hydrolysis in the presence of water or enzymes. When an ester bond is hydrolyzed, it undergoes chemical breakdown, resulting in the formation of the original carboxylic acid and alcohol. The objective of this study was to elucidate the impact of the simple suspension method and the crushing method of verapamil. To this end, we initially validated an in vitro dissolution test and subsequently conducted a pharmacokinetic test to compare various parameters (AUC_0–24_, area under the concentration-time curve; C_max_, maximum blood concentration; t_max_, time to reach the maximum blood concentration; t_1/2_, elimination half-life) of verapamil in healthy adults.

AUC is an index of how much of the drug was absorbed or is present in the body. The larger the AUC, the longer the drug remains in the body. C_max_ is used to evaluate the absorption rate of the drug and its distribution in the body. A high C_max_ indicates quick absorption of the drug. Finally, t_max_ is used to evaluate the absorption rate of the drug. A short t_max_ indicates that the drug is absorbed quickly.

## 2. Materials and Methods

### 2.1. Dissolution Test

#### 2.1.1. Test Method

A dissolution study was conducted on three types of processed test samples: verapamil hydrochloride tablets (sample 1), a simple suspension of verapamil hydrochloride tablets (sample 2), and crushed verapamil hydrochloride tablets (sample 3). For sample 1, one verapamil hydrochloride tablet was placed in a dissolution tester. Sample 2 was prepared in accordance with the “Parenteral and Enteral Nutrition Guidelines”, 3rd Edition [18]. In the human pharmacokinetics study, one tablet was placed between two sheets of paper, lightly tapped, and crushed into three to four grains with a pestle. The crushed tablet was then added to the medicine cup containing 20 mL of warm water at 55 °C and allowed to stand for 9.5 min. For the simple suspension method, it is recommended to use hot water at 55 °C [18], which maintains a temperature of 37 °C or higher for 10 min when left standing. This facilitates the dissolution of the medication. In accordance with the stipulations delineated in the Japanese Pharmacopoeia, the dissolution of a capsule is expected to occur within a 10-min timeframe when 50 mL of water is added, and the temperature is maintained at 37 °C ± 2 °C while shaking frequently. However, maintaining a precise temperature of 37 °C is challenging in practice. Consequently, the use of hot water at 55 °C in the simple suspension method is advised, given that 55 °C represents the minimum temperature at which the capsule remains above 37 °C, even if left standing for 10 min [18].

Subsequently, the medicine cup was agitated for 30 s and placed in the dissolution tester. Sample 3 was also prepared in accordance with the methodology employed in the human pharmacokinetics study. One tablet was sandwiched between two wrappers and crushed using a pestle and mortar, and it was visually confirmed that the tablets were uniformly crushed. One crushed tablet was then placed in the dissolution tester. One verapamil hydrochloride tablet was subjected to testing using the paddle method, with the following parameters: rotation speed of 50 rpm and test solution temperature maintained at 37.0 ± 0.5 °C. The volume of the test solution was 900 mL of water. Sampling of the solution was conducted at 2, 5, 15, 30, 60, and 90 min following the commencement of the test. However, for sample 3, 20 mL of warm water at 55 °C was added to the crushed tablets 10 min prior to the commencement of the dissolution test, thus forming a suspension.

The dissolution tester was a TOYAMA NTR-6400A (TOYAMA SANGYO Co., Ltd., Osaka, Japan) and the autosampler was a TOYAMA SAS-6000 (TOYAMA SANGYO Co., Ltd., Osaka, Japan). During the sampling process, 10 mL of eluate was collected, 10 mL of purified water was returned to the vessel, and the solution itself was maintained at 900 mL. Ten milliliters of the sampled eluate were passed through a 0.45 µm membrane filter and then analyzed as a sample using high-performance liquid chromatography (HPLC; SHIMADZU CORPORATION, Kyoto, Japan).

The HPLC analysis conditions were as follows: a YMC-PACK ODS-A(TOYAMA SANGYO Co., Ltd., Osaka, Japan) (150 × 4.6 mm i.d., S-5 μm, 12 nm Ser. No. 111GB30431) analytical column was used, the cell temperature was 40 °C, the flow rate was 1 mL/min, the measurement wavelength was 276 nm, and the mobile phase constituted methanol-water-perchloric acid at a ratio of 550:450:1.

A total of six vessels were prepared for each sample, and a release profile was generated by expressing the average elution rate for each period as a percentage.

Dissolution tests are correlated with several parameters of pharmacokinetic tests, as evidenced in previous studies. These tests are particularly closely related to AUC, C_max_, and t_max_. Based on these correlations, the outcomes of pharmacokinetic tests can be anticipated based on the results of dissolution tests. In the case of verapamil used in this study, it was unclear whether in vitro–in vivo correlation was established. Furthermore, the dissolution profile of dissolution test in water may differ from in vivo due to differences in the intragastric pH, which can change in solubility and absorption rates. Consequently, a range of pharmacokinetic parameters were examined through human pharmacokinetic tests.

#### 2.1.2. Study Drug

The verapamil hydrochloride 40 mg tablets (Japan Pharmacopoeia) used in this study were procured from Eisai Co., Ltd. (Vasolan^®^; serial number: 13A21K, Tokyo, Japan).

### 2.2. Subjects

This clinical study was conducted at the Showa University Clinical Research Institute for Clinical Pharmacology and Therapeutics and was approved by the Clinical Research Review Committee of the Showa University Educational Corporation (jRCTs031210270). This study included six participants who received comprehensive explanations, provided informed consent, and confirmed their eligibility. This study was conducted between September and October 2021.

Due to the menstrual cycle, women are subject to hormonal fluctuations that may affect the absorption, distribution, metabolism, and excretion of drugs. This may result in fluctuations in pharmacokinetic data, thereby complicating the analysis. Therefore, women were excluded from this study. Additionally, it has been established that the pharmacokinetics of verapamil differ by sex [19]. Consequently, only male subjects were included.

A total of six healthy adult males, aged between 20 and 40 years, were enrolled in this study. The screening tests, which included medical history, blood sampling, a history of alcohol consumption, a history of allergies, vital measurements, and electrocardiography, confirmed that the participants were healthy.

### 2.3. Eligibility

#### 2.3.1. Inclusion Criteria

- Age: to participate in the study, individuals had to be between 20 and 40 years at the time of consent.
- Sex: male
- Body mass index (BMI): between 18.5 and 28.5 kg/m^2^.
- It was imperative that participants provided informed consent, complied with research rules and regulations, underwent a preliminary examination outlined in the research protocol, and reported any subjective symptoms.
- The doctor responsible for monitoring the participant was obligated to determine whether the participant was healthy and eligible to participate in the research during the preliminary examination outlined in the research protocol.

#### 2.3.2. Exclusion Criteria

A meticulous examination of the screening test results was conducted to ascertain that Vasolan tablets were not administered to patients with severe congestive heart failure, atrioventricular block, or sinoatrial block of degree II or higher, or those with stiff bradycardia (less than 50 beats per min), WPW, or LGL syndrome. These conditions represent contraindications that are explicitly outlined in the package insert for Vasolan tablets. We further set the following exclusion criteria:

- Individuals with clinically significant electrocardiogram (ECG) abnormalities that may impact the safety of this study.
- Individuals with clinically problematic ECG abnormalities that may affect the safety of the study, a history of drug or alcohol abuse or dependence, or any heart, liver, renal, pulmonary, ocular, or hematological disease that may affect the evaluation and safety.
- Individuals who were currently taking any medications (including dietary supplements) at the time of enrollment that could affect the evaluation and safety of the study, as well as those who had a history of drug allergies.
- Individuals who had participated in another clinical trial within the past three months.
- Individuals who did not meet the eligibility criteria as determined by the investigator.

### 2.4. Study Design

**Figure 1:**
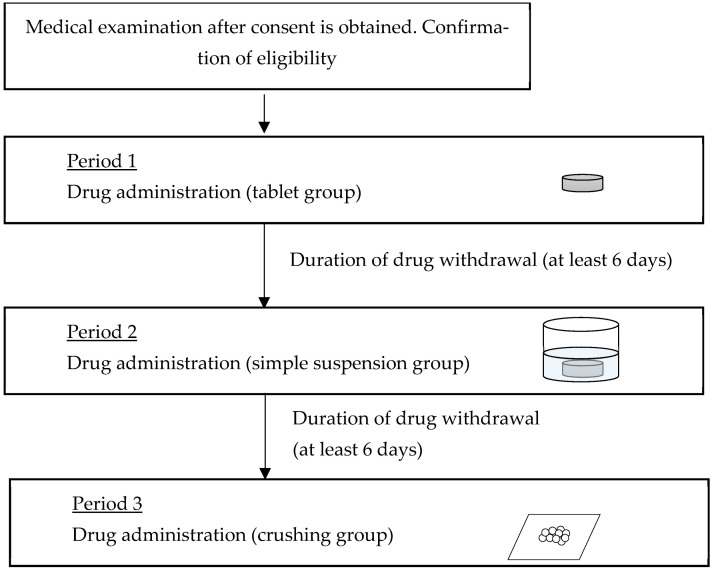
Schedule of the entire study.

The objective of this study was to examine the pharmacokinetics of three different formulations of the same drug administered to six study participants over three separate time periods. This study was conducted as a parallel-group comparison, with the tablet group serving as the control. Randomization was not performed to compare the amount of change in pharmacokinetic parameters due to different dosing methods for the same subjects. The administration schedule for the test formulations is illustrated in Figure 1. Following each dose, a six-day washout period was permitted for the drug to be metabolized and excreted. Blood drug levels were quantified in accordance with the study schedule and are presented in Table 1.

This study was conducted in accordance with the Declaration of Helsinki, the “Ethical Guidelines for Medical and Health Research Involving Human Subjects” and the “Clinical Research Law” and was approved by the Clinical Study Review Board of the Showa University Educational Corporation (jRCTs031210270). The principal investigator obtained written consent from the study subjects for their participation in this study.

Six participants were administered 40 mg of verapamil hydrochloride under fasting conditions, and the blood concentration of verapamil was measured over time. The drug was administered to the same participants under open-label conditions over three dosing periods (Figure 1): the first period (period 1) involved tablets (control group), the second period (period 2) involved a simple suspension, and the third period (period 3) involved crushed tablets (crushing group). The methodology employed for each study drug is illustrated in Figure 1.

In period 1 (tablet group), a 40-mg verapamil hydrochloride tablet was orally administered with water. In period 2 (simple suspension group), a 40-mg verapamil hydrochloride tablet was orally administered with water following processing using the simple suspension method. A six-day washout period was allowed between periods 1 and 2 and between periods 2 and 3 to allow for metabolic elimination of the drug. In the third period, a 40-mg verapamil hydrochloride was placed between two sheets of wrapping paper and crushed uniformly with a pestle on top of the wrapping paper. Upon crushing, the tablets were visually confirmed to have been crushed uniformly. Figure 1 illustrates the procedure.

### 2.5. Study Formulations

#### 2.5.1. Administration of Study Formulations

The participants were instructed to refrain from food and drinks after 9:00 p.m. on the day before drug administration for the entirety of the study period.

In the initial period (the tablet group), one 40-mg tablet of verapamil hydrochloride was orally administered directly with 100 mL of water. In the second period (simple suspension method group), the drug was prepared according to the *Handbook of Oral Drugs Administered by Tube—List of Medicines Capable of Simple Suspension Method*, 3rd Edition [18]. As stated in the instructions, tablets that do not disintegrate are to be lightly tapped to break the coating and facilitate disintegration and suspension. For verapamil hydrochloride, one tablet was placed between two sheets of paper, crushed with a pestle to 3–4 grains, placed in a medicine cup, and allowed to stand for 9.5 min after 20 mL of warm water at 55 °C was added. This procedure was conducted in accordance with the simple suspension method [18]. After 30 s of stirring the medicine cup, the subjects proceeded to consume the tablet suspension. Subsequently, an additional 80 mL of water was consumed, resulting in a total volume of 100 mL. In the third stage (crushing method group), the tablet was crushed on top of a sheet of wrapping paper using a pestle, ensuring that the tablet was sandwiched between the sheets of wrapping paper. The powders were visually inspected to ascertain that they had been uniformly crushed. Subsequently, the powder was suspended in 100 mL of water prior to administration.

#### 2.5.2. Blood Sampling and Observations

Blood samples for determining blood drug concentrations were collected on 11 occasions prior to drug administration and at 0.5, 1, 1.5, 2, 2.5, 3, 4, 6, 8, 10, and 24 h post-administration. At each designated sampling time, a total of 5 mL of blood was collected from the median cutaneous vein via the use of heparinized blood collection tubes. Each sample was promptly cooled and centrifuged (3000 rpm, 10 min), and the separated plasma was frozen at or below −20 °C until the drug concentrations were determined. To evaluate the pharmacokinetics of the study drug and its active metabolites and to ensure the safety of the study participants, the following data were recorded: sampling times, vital signs, physician visits, and adverse events.

### 2.6. Measurement of Blood Drug Concentration

The verapamil blood concentration was determined using liquid chromatography–electrospray ionization mass spectrometry (LC-ESI-MS). The LC and MS conditions were established in accordance with the findings of studies conducted by Chytil et al., Li et al., and do C Borges et al. [20,21,22]. All MS analyses were performed using a Prominence high performance liquid chromatography (HPLC) system (Shimadzu, Kyoto, Japan) equipped with a quadrupole linear ion trap mass spectrometer (QTRAP5500; Sciex, Framingham, MA, USA). Fifty microliters of plasma obtained from the study participants was supplemented with 50 µL of a 1:1 (*v*/*v*) solution of methanol in water, 100 µL of acetonitrile, and 50 µL of 1 mg/mL metoprolol in a 1:1 (*v*/*v*) methanol–water solution as an internal standard. The solution was vortexed for 10 s and then centrifuged at 16,000× *g* for 3 min at 4 °C. Subsequently, 50 μL of the supernatant was transferred to 150 μL of water, and 5 μL of the supernatant was quantified using LC-ESI-MS. Chromatographic separation was performed using an XTerra MS C18 Column (Waters Corporation; 186000446, Milford, MA, USA) at room temperature (10–25 °C). Mobile phase A consisted of 0.02% formic acid in acetonitrile, while mobile phase B was composed of formic acid (0.02%) in water. The gradient elution program was 95% to 50% solution B. The mobile phase was then subjected to a gradient elution program, with the composition changing as follows: 0–3.5 min, 50–5% solution B; 3.5–4 min, 5–95% solution B; 4–4.5 min, 95% solution B; and 4.5–7.5 min, 95% solution B. The eluate was introduced into the mass spectrometer at a flow rate of 0.3 mL/min. Verapamil was analyzed using a tandem quadrupole mass spectrometer via multiple reaction monitoring (MRM) in the positive-ion mode. The *m*/*z* transitions monitored were as follows: mass-to-charge ratios were used to identify verapamil and metoprolol (455.40/165.20 for verapamil and 268.50/116.20 for metoprolol). Verapamil was identified in the samples by matching their MRM signals and LC retention times to those of a pure standard. Verapamil was quantified using standard curves of the analyte relative to the internal standard.

### 2.7. Calculation of Pharmacokinetic Parameters

The primary endpoints were the pharmacokinetic parameters AUC_0–24_ and C_max_, which were calculated. The t_max_ and t_1/2_ were calculated as secondary endpoints. AUC_0–24_ was calculated using the trapezoidal method based on actual measurements. C_max_ and t_max_ were calculated using the JMP^®^ Pro 16.2.0 software (SAS Institute, Japan, Tokyo) with actual measurements. The t_1/2_ was calculated using MOMENT (Microsoft Excel^®^ 16) by dividing ln2 by the terminal disappearance rate constant λ, which was calculated using linear regression analysis of data at four or more points in the terminal portion.

### 2.8. Statistical Analysis

#### 2.8.1. Methods of Statistical Analysis

The results were analyzed for all study participants, except for those who did not complete the endpoint measurement.

The changes in the pharmacokinetic values obtained in periods 2 (simple suspension group) and 3 (crushing group) were compared using period 1 (tablet group) as the control. Continuous variables are expressed as the mean ± standard error. The significance level was set at *p* < 0.05, and statistical analyses were conducted using the JMP^®^ Pro 16.2.0 software. The test was conducted after logarithmic conversion of the measured values for AUC_0–24_, C_max_, and t_1/2_ in accordance with the bioequivalence test guidelines for generic drugs issued by the MHLW. The log-transformed values of AUC_0–24_ and C_max_ of verapamil in the blood were subjected to a repeated-measures analysis of variance (ANOVA) in three periods. When the p-value was less than 0.05, multiple comparisons were performed using Tukey–Kramer post hoc tests. Furthermore, the distribution of the measured values was evaluated for normality using t_max_. In the event that a non-normal distribution was identified (*p* < 0.05), the three groups were compared using the Friedman test. As this study was a pilot study, the level of statistical significance was set at 5%.

The statistical analysis was conducted using JMP^®^ Pro 15.0.0 (SAS, Tokyo, Japan).

#### 2.8.2. Safety Evaluation

Any subjective symptoms or other findings observed prior to or following the administration of the study drug, or any abnormal vital signs, were considered adverse events. The degree and relevance of these events to the study drug were then determined.

## 3. Results

### 3.1. Dissolution Test

**Figure 2:**
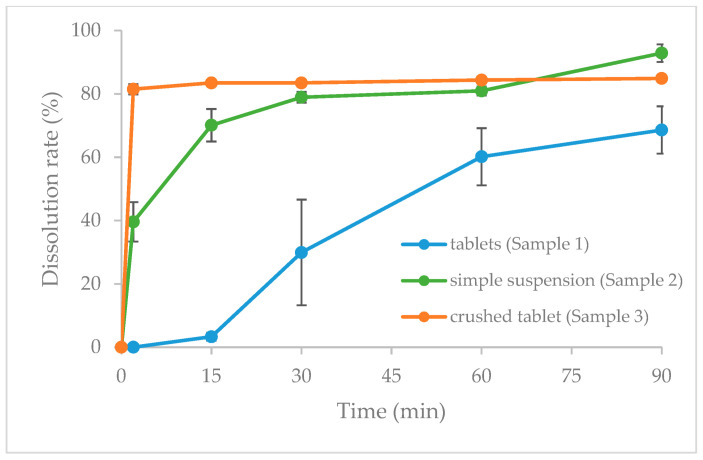
Dissolution rate of verapamil tablet (sample 1); simple suspension of verapamil hydrochloride tablets (sample 2); and crushed verapamil tablet (sample 3); mean ± SD, *n* = 6.

Figure 2 illustrates the change in dissolution rate over time for verapamil hydrochloride tablets (sample 1), verapamil hydrochloride tablets in simple suspension (sample 2), and crushed verapamil hydrochloride tablets (sample 3).

The dissolution test results demonstrate that the dissolution profiles of the tablets and the crushed product were distinct. In the tablets (sample 1), verapamil hydrochloride was undetectable 2 min after the commencement of the test, and dissolution was observed from 15 min onward, followed by a gradual increase in the dissolution rate. Conversely, for the simple suspension product (sample 2), the dissolution rate was only 39.57% after 2 min, but after 15 min, the rate increased to 70.11%. Notable results were obtained for the crushed product (sample 3), with a dissolution rate of 81.48% after 2 min.

### 3.2. Subjects

This in vivo study included six participants, none of whom discontinued or withdrew from of the study. The study participants were 32.2 ± 4.3 years of age and had a BMI of 24.3 ± 2.7 kg/m^2^.

### 3.3. Blood Concentration

**Figure 3:**
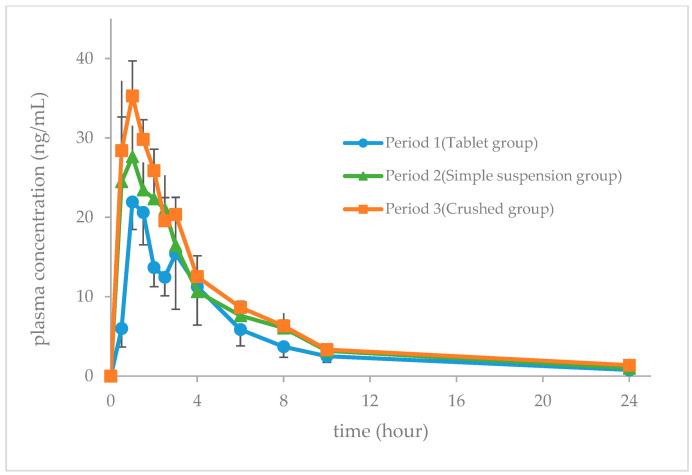
Blood verapamil concentration after administration (tested in different participants; mean ± SE, n = 6).

The mean concentration-time trends of verapamil hydrochloride during the three periods are presented in Figure 3. The mean AUC_0–24_, C_max_, t_max_, and t_1/2_ values for the blood concentration of verapamil are presented in Table 2. The methodology employed in this study has previously been subjected to rigorous validation procedures [20,21,22].

The sample size was six, with the mean ± standard error presented. The area under the concentration-time curve (AUC_0–24_) was calculated, as was the maximum blood concentration (C_max_), the time to reach the maximum blood concentration (t_max_), the elimination half-life (t_1/2_), the elimination rate constant (Kel), and the mean residence time (MRT). The significance level was set at *p* < 0.05. For the purposes of this study, logarithmically converted values were tested using repeated-measures ANOVA over the three periods for the log-transformed values of AUC_0–24_, C_max_, and t_1/2_. When the *p*-value was less than 0.05, multiple comparisons were performed using the Tukey–Kramer post hoc test. Furthermore, the time to maximum concentration (t_max_) was determined over the three periods using the Friedman test.

### 3.4. Safety Endpoints

No adverse events were observed throughout the study period. No abnormalities were observed in subjective symptoms or other findings.

## 4. Discussion

The objective of this study was to evaluate the pharmacokinetics of verapamil in healthy volunteers following processing using the crushing and simple suspension methods. The area under the curve over the first 24 h and the maximum concentration of verapamil increased in the simple suspension group and were significantly higher in the crushing group than in the tablet group.

In the case of administering food through a nasogastric or gastric tube, it is common practice to administer medication through the same tube. However, tablets cannot pass through the tube; therefore, they are replaced with liquid formulations. Nevertheless, in instances where liquid or powder dosage forms are not available, it is recommended that pharmacists consider administering the drug via a tube [4]. A survey of 753 hospitals across Japan revealed that 98% of hospitals administered drugs through a tube, with 78% selecting the simplified suspension method as the method of drug administration [23]. The crushing of tablets confers the advantage of enabling the administration of medications through feeding tubes, thereby facilitating the process of swallowing for patients with dysphagia, although it could also change the pharmacokinetic behavior of the medication as shown in the current study.

The simple suspension method dissolves drugs in warm water without crushing them, significantly shortening the pharmacist’s work time and preventing drug loss. This method also maintains the stability of the drug and improves the work efficiency of pharmacists and nurses [11,24]. The stability of drugs processed using the simple suspension method has been investigated in several in vitro studies. In the case of zonisamide, ticlopidine, and warfarin, the active pharmaceutical ingredient was found to be stable when suspended in warm water at 55 °C and left for 2 h at room temperature [8].

In contrast, the Vasolan^®^ tablet did not disintegrate when placed directly in a syringe and left freely for 5 min with 20 mL of warm water at 55 °C. Furthermore, the tablet did not disintegrate when suspended for another 5 min. Subsequently, the syringe was then agitated by rolling it back and forth 15 times to facilitate the disintegration of the tablet. Consequently, when administering Vasolan^®^ tablets via the simple suspension method, it is advised that one tablet be encased in wrapping paper, tapped on the top with a pestle to break it, and then left in a syringe containing 20 mL of warm water at 55 °C for 5 min. Subsequently, the syringe should be agitated by rolling back and forth 15 times to disintegrate and suspend the tablet [18]. Verapamil, the active ingredient of Vasolan^®^, photodegrades in aqueous solutions, resulting in the cleavage of C-N bonds in the amine moiety. In the case of administering Vasolan^®^ tablets to patients with dysphagia, failure to achieve the expected efficacy of the drug by ensuring accurate delivery will not only be detrimental to the patient but a waste of limited medical resources. The objective of this study was to investigate the dosing and pharmacokinetics of 40 mg verapamil hydrochloride tablets in healthy adult males with preserved physiological function. To this end, three different dosing methods were employed as follows: the method described in the package insert, the simple suspension method, and the crushing method.

A single oral dose of 80 mg of verapamil in ten healthy adult males was reported to result in an AUC_0–24_ of 450.9 ng·h/mL, C_max_ of 86.2 ng/mL, and t_max_ of 2.2 h [17]. Although direct comparison of the pharmacokinetic parameters obtained in this study with those obtained with 80 mg verapamil was challenging, the AUCs in the current study were approximately 50% of those obtained with 80 mg verapamil during each period; thus, the pharmacokinetics obtained herein were considered reliable.

In a previous study by Yano et al., the recovery rate of the primary drug was found to be reduced when the crushing method was employed [8]. The authors postulated that the main drug may have adhered to the pestle and mortar, resulting in a reduction in the recovery rate. Consequently, a dispensing method was devised in this study that minimized drug adhesion to the dispensing device, and the tablets were prepared in such a way that there was minimal or no loss of drug in a single dose. Aqueous solutions of verapamil are susceptible to photodegradation [7], which may result in drug degradation when verapamil hydrochloride is mixed with water prior to administration. In the current study, the test drug was only mixed with water after it was placed in the mouth. Consequently, the potential change induced by the placement of verapamil in an aqueous solution was deemed negligible. However, the AUC_0–24_ of verapamil was 133.4% in the second period (simple suspension method group) and 179.1% in the third period (crushing group). The C_max_ was 150.5% in the second period (simple suspension method group) and 171.3% in the third period (crushing group), indicating that the AUC and C_max_ of verapamil increased with both methods.

This phenomenon can be attributed to two factors: the dissolution rate and the decay rate. In general, it is anticipated that powders exhibit a more rapid onset of action than tablets or capsules. This is due to the significant influence of particle size and surface area on drug solubility. The Nernst–Noyes–Whitney equation [25] states that if the dissolution process is driven by diffusion, the dissolution rate (dC/dt) can be expressed as follows:

dC/dt = (D · S) · (Cs − C)/(V · δ),

where D = diffusion coefficient, S = specific surface area, V = solution volume, δ = diffusion layer thickness, Cs = solubility, and C = internal solution concentration. Consequently, an increase in surface area will result in a faster dissolution rate. The enhanced porosity surrounding a compressed tablet allows for the penetration of water into the internal voids, thereby expanding the effective surface area within the tablet. This is derived from the extended Nernst–Einstein–Whitney equation [26].

An increase in the surface area of the drug results in a faster dissolution rate. However, the dissolution process is accelerated under sink conditions due to the influence of the particle size on the C value [27]. Similarly, for poorly soluble drugs, such as barium sulfate, oxazepam, and glibenclamide, particle shape and size have been demonstrated to influence dissolution rates, with a reduction in dissolution rates observed as particle size increases [28].

It has been postulated that in the crushing method group (period 3), the AUC and C_max_ increased in comparison to those in the tablet group (period 1). This is attributed to the alteration in the release behavior of verapamil, resulting from the larger surface area in contact with water and the smaller particle size due to the crushing of the tablets. In the simple suspension method group (period 2), it is important to note that the effect of temperature and the change in particle size due to tablet crushing cannot be disregarded. In the simple suspension method, the crushed tablets were immersed in warm water at 55 °C for approximately 10 min. The rise in temperature led to an increase in the diffusion coefficient (D) and dissolution rate (dC/dt). Consequently, it was postulated that the release profile of verapamil underwent a transformation, resulting in augmented AUC and C_max_ values.

The dissolution test results indicated that the tablets (sample 1) underwent dissolution after 15 min from the commencement of the test, with the dissolution rate increasing gradually after 30 min. Conversely, the crushed product (sample 2) exhibited a dissolution rate of 81.48% after 2 min. In the simplified suspension (sample 3), drug release after 2 min was only 39.57%, but increased to 70.11% after 15 min. Overall, the dissolution experiments demonstrated that the release profiles differed for the three different methods of administration: tablets, crushed product, and simple suspension.

For a drug to be absorbed in vivo after oral administration, it must disintegrate and dissolve in water or other solvents. The elevation in C_max_ and AUC observed in this study were significantly influenced by the drug’s dissolution profile. Nevertheless, it is essential to exercise caution when interpreting the results of the dissolution studies. This is because in vivo, in addition to the solubility of the drug product, other factors such as low bioavailability may contribute to the elevation. The methodology employed to quantify verapamil plasma concentrations in this study has previously been validated [20,21,22]. As sufficient validation was conducted prior to drug concentration measurement in this study which assured the quality of the data. Consequently, it is anticipated that the measurement method used in this study had comparable reliability.

The pharmacokinetics of drugs may be affected by simple suspension and crushing administration. The use of the simple suspension method has been demonstrated to affect the rate of absorption and maximum blood concentration of drugs, as well as alter the bioavailability of certain drugs. Lansoprazole suspensions exhibited a shorter and higher C_max_ than capsules yet demonstrated lower overall drug availability (AUC) [29]. Crushed prasugrel was absorbed three times faster than the whole tablets, thus exhibiting a more rapid and potent antiplatelet effect [16,30]. The dissolution of dolutegravir/abacavir/lamivudine (TRI) resulted in increased exposure and an improved absorption rate of dolutegravir [31], indicating that the process of crushing a drug enhances its absorption rate. Crushing pentoxifylline also demonstrated a higher C_max_ and shorter t_max_ than those from the intact tablets. However, this resulted in an increased incidence of dose-dependent adverse effects [32]. By contrast, crushed pazopanib exhibited increased AUC and C_max_ values, along with an enhanced absorption rate, when compared to intact tablets [33]. It can be reasonably deduced that the processing of a drug prior to its administration often affects its absorption rate and bioavailability, particularly in instances where the absorption rate is increased, and drug exposure is elevated. It is therefore important to exercise caution when evaluating the characteristics of the drug and the condition of the patient when considering the use of simple suspension or crushing methods.

Regarding the disintegration rate, the *Handbook of Oral Drug Administration by Tube*, 4th Edition, indicates that Vasolan^®^ tablets can be administered by tube by breaking the tablet coating. Subsequently, the tablet disintegrates and suspends within 10 min and is passed through an 8 Fr tube or gastro bottle [18]. During our study, the Vasolan^®^ tablets were disintegrated in a medicine cup prior to administration in the simple suspension method group (period 2). Disintegrants are frequently incorporated into common tablets with the objective of dispersing the tablets into individual particles and facilitating their absorption. Hydroxypropyl cellulose and corn starch were utilized as additives in the Vasolan^®^ tablets employed in this study. In the simple suspension method employed in this study, the tablets were pre-broken, thereby facilitating the penetration of water through the tablet surface and interior pores, which is believed to markedly enhance the disintegration rate in comparison to that of conventional tablets. Consequently, the time required for the tablets to disintegrate in the stomach was reduced, and the time to reach the maximum blood concentration, t_max_, was shorter in the crushing (3rd period) and simple suspension (2nd period) method than in the tablet group (1st period). In elderly patients and patients with impaired liver or kidney function, delayed drug metabolism and excretion can result in an increased AUC and C_max_, with the potential for the drug to remain in the body for an extended period. It is of concern that an increase in drug concentration may result in an elevated risk of adverse effects. Accordingly, in patients with these backgrounds, it is imperative to select the appropriate drug, adjust the dosage, and monitor pharmacokinetics. The administration of verapamil via the simple suspension and crushing methods also resulted in an increased AUC and C_max_. It is imperative to exercise caution, as a multitude of factors may precipitate a surge in AUC and C_max_ that exceeds expectations.

Verapamil is a calcium antagonist that exerts a negative inotropic effect [17], reducing the contractile force of the myocardium. It is common for elderly individuals to experience a decline in cardiac function, and the administration of verapamil in a crushed or suspended form may exacerbate heart failure. Furthermore, elderly individuals may be more prone to develop bradycardia and atrioventricular block. Furthermore, elderly individuals exhibit a diminished capacity to regulate blood pressure. Consequently, an elevation in the AUC and C_max_ of verapamil may precipitate a decline in blood pressure. This increases the risk of dizziness and fainting. Furthermore, elderly individuals frequently take multiple medications [34,35,36], which may result in enhanced adverse effects due to interactions with verapamil. Caution is required when using it in combination with drugs that inhibit the CYP3A4 enzyme or other cardiovascular drugs, as this may result in enhanced adverse effects [37,38].

Crushing and simple suspension methods are safe and straightforward for patients who have difficulty ingesting or swallowing or are on tube feeding. Nevertheless, the processing of pharmaceuticals prior to administration may result in the emergence of unintended side effects or an increase in the prevalence of side effects. Eisai Co., Ltd., the manufacturer and distributor of Vasolan^®^ tablets utilized in this study, also provides information for healthcare professionals on its website regarding the simple suspension method. This information states the following: “The administration of the drug in suspension is not an approved method of administration, and we do not recommend administration of the drug in suspension”. As the drug has been approved for administration in its original form, there has been no study of its efficacy and safety in suspension [39]. The company states the following: “The results of this study are based on the results of a safety study conducted by Eisai Co.”. Our study results corroborate the safety concerns of Eisai Co.

Previously, we reported on the effects of alterations in administration methods, such as crushing and simple suspension, on pharmacokinetics. In a study on healthy adults, the simple suspension method resulted in lower AUC_0–24_ and lower C_max_ due to the difference in reactivity of degradation of temocapril degradation by magnesium oxide, which affected the pharmacokinetics [40]. The observed variation in pharmacokinetic parameters in this study differs from that observed for the combination of temocapril and magnesium oxide. This discrepancy is attributed to differences in the solubility of verapamil hydrochloride in the simple suspension and crushing methods, which in turn results in differences in pharmacokinetics between the simple suspension and crushing methods. Although these two studies suggest that the suppression of the dissolution of the main drug may be a factor, there is no generalizable method at this time. Therefore, it is necessary to consider a method of administration that minimizes the impact on drug pharmacokinetic parameters.

A limitation of this study is that the participants were healthy adults, and a group of patients with diseases who should be taking verapamil hydrochloride was not used as a control. Consequently, it was not possible to evaluate the extent to which the variations in AUC_0–24_ and C_max_ affect drug efficacy. Furthermore, physiological functions such as hepatic metabolism and renal excretion are impaired in older adults and in patients with feeding and swallowing disorders. Consequently, the results of this study cannot be generalized to other patient cohorts, and caution should be exercised during application. Furthermore, the study was limited by a focus on healthy male volunteers, which raises questions about the generalizability of the findings. In particular, drugs such as L-dopa, aspirin, and capecitabine have been observed to undergo chemical decomposition in alkaline solutions or under fluctuating pH conditions. It should be noted that the results of this study may not be applicable to all drugs [11]. Furthermore, as this study was a pilot study, the sample size was relatively small, which may have resulted in a reduction in statistical power and limited generalizability. In the future, the results of this study should be confirmed by an evaluation of pharmacokinetics in patients who actually take verapamil using the simple suspension method and the crushing method, as well as in elderly patients. The objective of this study was to compare the effects of the simple suspension method to those of the crushing methods on the pharmacokinetics of verapamil. The results demonstrated that the AUC_0–24_ and C_max_ values of verapamil were significantly elevated in the crushing group in comparison to the other groups. These findings may be attributed to alterations in the dissolution and release rates resulting from the processing of the drug prior to administration. Given the possibility that the simple suspension method may also result in an increased AUC_0–24_ and C_max_, it is important to monitor fluctuations in vital signs during use. It is our hope that the results of this study will assist in the selection of appropriate drug administration methods for patients with feeding and swallowing difficulties.

## Figures and Tables

**Table 1:** Schedule for research implementation.

Elapsed Time (h)	Pre	0	0.5	1	1.5	2	2.5	3	4	5	6	8	10	24
Vital signs measurement	X			X		X			X			X		X
Medical examination	X			X		X			X					X
Blood sampling	X		X	X	X	X	X	X	X	X	X	X	X	X
Drug administration		X												
Meal									X *			X *		X *
Subjective symptom survey	X	Anytime												
Adverse event observation		Anytime												

The X in the table indicates the timing of the inspection. * Meals were allowed once blood sampling was completed.

**Table 2:** Pharmacokinetic parameters.

	Pharmacokinetic Parameters			Post-Test *p*-Value		
	1st Period (Tablet Group)	2nd Period (Simple Suspension Group)	Period 3 (Crushing Group)	Period 1–2	Period 1–3	Period 2–3
AUC_0–24_ [mg-h/L]	116.21 ± 31.06	155.08± 28.96	208.15 ± 35.75	1.46 [0.85, 2.52] 0.184	1.99 [1.16, 3.43] 0.015 *	1.36 [0.79, 2.35] 0.304
C_max_ [mg/L]	26.47 ± 5.09	39.83 ± 10.29	45.34 ± 9.79	1.48 [0.91, 2.41] 0.116	1.80 [1.11, 2.93] 0.019 *	1.22 [0.75, 1.98] 0.535
t_max_ [mg-h/L]	1.50 ± 0.32	1.08 ± 0.20	1.08 ± 0.08	-	-	-
t_1/2_ [h]	7.87 ± 0.61	7.14 ± 0.82	7.57 ± 0.28	-	-	-
Kel	0.091 ± 0.007	0.110 ± 0.015	0.092 ± 0.003			
MRT	2.75 ± 0.06	2.72 ± 0.08	2.64 ± 0.10			

Statistical significance between each period was indicated by an asterisk (*) for *p* < 0.05.

## Data Availability

All relevant data are included in the article.
